# Effects of body mass index on mortality in elderly patients with hip fractures

**DOI:** 10.1097/MD.0000000000039157

**Published:** 2024-08-02

**Authors:** Chan-Hee Park, Seung-Hoon Lee, Rim Lee, Dong-Young Kim, Myung-Rae Cho, Suk-Kyoon Song

**Affiliations:** a Department of Surgery, School of Medicine, Keimyung University and Dongsan Medical Center, Daegu, Republic of Korea; b School of Medicine, Daegu Catholic University, Daegu, Republic of Korea; c Department of Orthopaedic Surgery, Gumi Hyundai Hospital, Gumi, Republic of Korea; d Department of Orthopaedic Surgery, School of Medicine, Daegu Catholic University Medical Center, Daegu, Republic of Korea.

**Keywords:** body mass index, hip fracture, mortality

## Abstract

Hip fractures remain a substantial health concern, particularly among elderly individuals with osteoporosis, leading to high global mortality rates. This study aimed to analyze the association between body mass index (BMI) and postoperative mortality in patients who underwent surgery for hip fractures. A total of 680 patients treated at a single institution between January 2018 and December 2022 were included. Factors such as age, BMI, sex, Charlson Comorbidity Index (CCI), preoperative hemoglobin levels, American Society of Anesthesiologists score, anesthesia method, duration of surgery, and time from injury to surgery were assessed. Underweight status, male sex, higher CCI, and general anesthesia were significantly associated with 1-year and in-hospital mortality. Notably, underweight individuals exhibited a higher risk of mortality than normal-weight individuals, and female patients had lower mortality rates. This study underscores the importance of considering BMI, along with other demographic and clinical factors, in predicting postoperative mortality among patients with hip fractures, aiding the development of tailored management strategies to improve outcomes and reduce complications in this vulnerable patient population.

## 1. Introduction

Hip fracture is a significant health concern that is particularly frequent among the elderly population with osteoporosis, with high mortality rates of up to 22%.^[1,2]^ Approximately 14 million patients worldwide experience hip fractures yearly,^[3]^ and >30,000 patients in Korea suffer from hip fractures annually.^[4]^ Despite recent studies showing a gradual decrease in mortality in those patients with hip fractures,^[1,5]^ the incidence of hip fractures is expected to increase,^[5]^ warranting continuous attention. Moreover, the postoperative recovery process in elderly patients is often prolonged and complex, with various complications, such as pneumonia, urinary tract infections, and venous thromboembolism commonly observed.^[6]^

Studies have reported that factors such as old age, male sex,^[7]^ and delayed surgery from hospital admission >48 hours^[8]^ are associated with higher mortality rates. Moreover, it is well-established that patients with a high or low body mass index (BMI) have an increased risk of complications following orthopedic surgery compared to those with normal BMI. Obesity not only increases mortality after surgery but also increases the risk of complications, including acute renal failure and higher rates of surgical site infections, whereas underweight patients have higher rates of perioperative transfusions.^[9,10]^ However, few studies have focused on hip fractures, specifically comparing mortality between overweight and underweight individuals.

This retrospective study aimed to analyze the association between BMI and postoperative mortality in elderly patients with hip fractures. We hope to facilitate perioperative management and treatment strategies based on BMI in elderly patients with hip fractures, contributing to improved mortality rates and reduced complications in elderly patients undergoing hip fracture surgery.

## 2. Methods

### 2.1. Ethical considerations

This trial was approved by the Institutional Review Board of Daegu Catholic University Medical Center and was in accordance with local ethical guidelines. A Waiver of Documentation of Consent was granted by the Institutional Review Board because this was a retrospective study based on medical records, and the data obtained were protected in secured storage. This study also had no possibility of benefitting or harming the patients involved.

### 2.2. Study description and definitions

**Figure 1.**
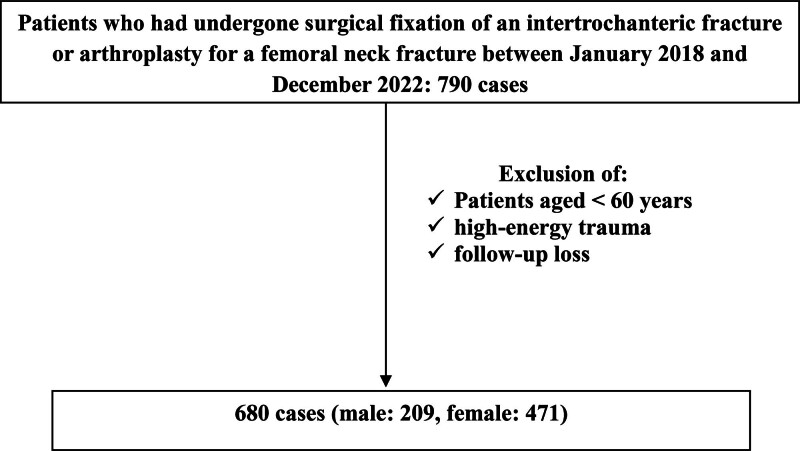
Flow chart of patients with hip fracture.

We conducted a retrospective study of patients who underwent surgical fixation of an intertrochanteric fracture or arthroplasty for a femoral neck fracture by a single surgeon at our hospital between January 2018 and December 2022. The study included 680 of 790 patients who underwent surgery between 2018 and 2022. Patients aged <60 years, those with high-energy trauma, and those who were not followed up were excluded (Fig. 1). Previous research on patients with hip fractures has commonly classified individuals aged ≥60 years as geriatric patients.^[11–13]^ These studies have demonstrated that being >60 years of age is a significant factor independently associated with higher mortality rates and increased postoperative complications.^[14,15]^ In our study, we defined geriatric patients as those aged ≥60 years, utilizing the same criteria for classification.

BMI was calculated as weight in kilograms divided by height in meters squared (kg/m^2^). The BMI classification was defined according to the WHO Asia Asia-Pacific regional guidelines, and we used the following cutoff points to divide the participants into three groups according to BMI category: underweight (<18.5 kg/m^2^), normal weight (18.5–24.9 kg/m^2^), and overweight/obese (≥25.0 kg/m^2^).^[16–19]^ We studied the demographics and clinical outcomes of elderly patients with hip fractures and the impacts of age and BMI on mortality.

### 2.3. Data collection

The following data were collected retrospectively: patient characteristics, including age, sex, BMI, Charlson Comorbidity Index (CCI), and American Society of Anesthesiologists (ASA) score; and clinical data, including laboratory data at the admitted emergency room, method of anesthesia, type of operation, postoperative complications, duration of surgery, time from injury to operation, length of hospital stay, in-hospital mortality, and 1-year mortality.

### 2.4. Mortality analyses

We hypothesized that patient age, BMI, sex, CCI score, preoperative hemoglobin level, ASA score, method of anesthesia, duration of surgery, and time from injury to surgery would affect mortality. These data were collected from the electronic medical records. In all patients, a 2 L oxygen nasal cannula was routinely used after surgery, and oxygen was removed on the second day unless otherwise noted. Medical staff and caregivers provided training and performed chest percussions for expectorated sputum. In all the patients, thromboembolic prophylaxis was administered via pneumatic compression pumps during hospitalization. A Foley catheter was placed during surgery in the operating room, and removal was performed in batches on postoperative day 2.

### 2.5. Statistical analyses

Continuous data are expressed as the mean ± standard deviation. Categorical data are expressed as frequencies and percentages. Continuous variables were analyzed using Student *t* test or the Mann–Whitney *U* test, whereas categorical variables were analyzed using the chi-square or Fisher’s exact test. Univariate and multivariate logistic regression analyses were performed to identify the risk factors for 1-year and in-hospital mortality, with the degree of association presented as odds ratios and their corresponding 95% confidence intervals. Logistic regression performance was evaluated using C statistics and Hosmer–Lemeshow tests. Statistical significance was set at a *P* value < .05. The Statistical Package for the Social Sciences software (version 28.0; IBM Corporation, Armonk) was used for all analyses.

## 3. Results

### 3.1. Demographic characteristics

Table 1 presents the general characteristics of the participants. A total of 680 patients underwent surgery for hip fracture over a 5-year period. The participants had a mean age of 78.6 ± 8.4 (range 60–99) years. The majority of patients in this study were female (69.3%). There were 202 (29.7%) underweight (BMI < 18.5 kg/m^2^), 365 (53.7%) normal-weight (BMI 18.5–24.9 kg/m^2^), and 113 (16.6%) overweight/obese (BMI ≥ 25 kg/m^2^) participants. The mean CCI was 4.1 ± 1.2. The ASA score of the patients was mostly 2 (54.1%) and 3 (30.7%), with scores of 1 and 4 in 12.5% and 2.6% of the participants, respectively. The mean preoperative hemoglobin level was 11.0 ± 1.8 g/dL, and the mean albumin level was 3.7 ± 0.5 g/dL. A total of 244 patients received general anesthesia, and 436 received spinal anesthesia. There were 407 intertrochanteric fractures (59.9%) and 273 femoral neck fractures (40.1%). Postoperative complications included pneumonia in 229 patients (33.7%), pulmonary thromboembolism in 62 patients (9.1%), and urinary tract infection in 63 patients (9.3%). Regarding differences in postoperative complications among weight groups, there were no statistically significant differences in the rates of pneumonia (34.5% vs 32.2% vs 26.2%, *P* = .235), PTE (10.5% vs 7.9% vs 6.9%, *P* = .405), and UTI (9.6% vs 7.5% vs 9.0%, *P* = .612). However, the incidence rates were highest in the underweight group compared to the normal weight and overweight groups. The mean duration of surgery was 138.6 ± 38.1 minutes, and the time from injury to operation was 134.5 ± 137.3 hours. The mean length of hospital stay was 24.5 ± 13.8 days. The in-hospital mortality rate was 5.0% (n = 34/680), and the 1-year mortality rate was 12.5% (n = 85/680). Additionally, in-hospital mortality (8.3% vs 3.4% vs 2.1%, *P* = .005) and 1-year mortality (16.2% vs 10.1% vs 8.3%, *P* = .028) were significantly higher in the underweight group compared to the normal weight and overweight groups.

**Table 1:** Basic characteristics of all participants.

Variable	N = 680
Age (yr)	78.6 ± 8.4
Sex	
Male	209 (30.7)
Female	471 (69.3)
Body mass index (kg/m^2^)	22.0 ± 3.6
Underweight (BMI < 18.5)	202 (29.7)
Normal weight (18.5 ≤ BMI < 25)	365 (53.7)
Overweight and obese (25 ≤ BMI)	113 (16.6)
Charlson Comorbidity Index	4.1 ± 1.2
ASA score	
1	85 (12.5%)
2	368 (54.1%)
3	209 (30.7%)
4	18 (2.6%)
Preoperative hemoglobin (g/dL)	11.0 ± 1.8
Preoperative albumin (g/dL)	3.7 ± 0.5
Anesthesia	
General	244 (35.9%)
Spinal	436 (64.1%)
Operation type	
Osteosynthesis	407 (59.9%)
Total hip arthroplasty	198 (29.1%)
Bipolar hemiarthroplasty	75 (11.0%)
Postoperative complication	
Pneumonia	229 (33.7%)
PTE	62 (9.1%)
UTI	63 (9.3%)
Duration of surgery (min)	138.6 ± 38.1
Time from injury to operation (h)	134.5 ± 137.3
Length of hospital stay (d)	24.5 ± 13.8
In-hospital mortality	34 (5.0%)
One-year mortality	85 (12.5%)

ASA = American Society of Anesthesiologists, PTE = pulmonary thromboembolism, UTI = urinary tract infection.

### 3.2. Mortality

Univariate and multivariate logistic regression analyses identified underweight, male sex, higher CCI, general anesthesia, and time from injury to operation as factors related to in-hospital mortality. In-hospital mortality was significantly associated with the underweight group in the multivariate logistic analysis, and the risk was approximately 2.58 times higher than that in the normal-weight group (*P* = .014). In-hospital mortality was approximately 0.32 times lower in female patients than in male patients (*P* = .002) and increased by approximately 1.32 times for a CCI increase of 1 (*P* = .046). In-hospital mortality was approximately 2.30 times higher in those who received general anesthesia patients than in those who received spinal anesthesia (*P* = .029; Table 2).

**Table 2:** Univariate and multivariate logistic regression analyses for variables significantly associated with in-hospital mortality.

Variable	Univariate analysis		Multivariate analysis	
	OR (95% CI)	*P* value	OR (95% CI)	*P* value
Age (yr)	1.03 (0.99–1.08)	.171		
BMI group (ref: normal)				
Underweight (BMI < 18.5 kg/m^2^)	2.30 (1.11–4.78)	.025*	2.58 (0.84–1.04)	.014*
Overweight and obese (25 kg/m^2^ ≤ BMI)	0.68 (0.19–2.42)	.556	0.48 (0.12–2.02)	.319
Female	0.37 (0.19–0.75)	.005*	0.32 (0.15–0.65)	.002*
Charlson Comorbidity Index	1.33 (1.00–1.77)	.047*	1.32 (1.01–1.83)	.046*
Preoperative hemoglobin	1.02 (0.84–1.24)	.834		
ASA score (ref: 1)				
2	2.13 (0.49–9.38)	.315		
3	2.75 (0.61–12.47)	.189		
4	2.44 (0.21–28.47)	.476		
General anesthesia	2.09 (1.05–4.18)	.037*	2.30 (1.09–4.86)	.029*
Duration of surgery (min)	1.00 (0.99–1.01)	.645		
Time from injury to operation (h)	1.00 (1.00–1.00)	.012*	1.00 (1.00–1.00)	.007*

ASA = American Society of Anesthesiologists, BMI = body mass index, CI = confidence interval, OR = odds ratio.

* *P* ≤ .05 was considered to indicate statistical significance.

Underweight, male sex, higher CCI, and higher ASA were associated with 1-year mortality in univariate and multivariate logistic regression analyses. One-year mortality was significantly associated with the underweight group in the multivariate logistic analysis, and the risk was approximately 1.68 times higher than that in the normal-weight group (*P* = .044). One-year mortality was approximately 0.44 times lower in female patients than in male patients (*P* = .001) and increased by approximately 1.30 times for a CCI increase of 1 (*P* = .009). One-year mortality was 3.26 and 3.16 times higher in those with ASA = 2 than in those with ASA = 1 in the univariate (*P* = .027) and multivariate (*P* = .034) logistic regression analysis, respectively (Table 3).

**Table 3:** Univariate and multivariate logistic regression analyses for variables significantly associated with 1-year mortality.

Variable	Univariate analysis		Multivariate analysis	
	OR (95% CI)	*P* value	OR (95% CI)	*P* value
Age (yr)	1.01 (0.99–1.04)	.395		
BMI group (ref: normal)				
Underweight (BMI < 18.5 kg/m^2^)	1.64 (1.00–2.69)	.048*	1.68 (1.01–2.79)	.044*
Overweight and obese (25 kg/m^2^ ≤ BMI)	0.88 (0.43–1.77)	.713	0.84 (0.41–1.71)	.628
Female	0.50 (0.31 - 0.79)	.003*	0.44 (0.27–0.71)	.001*
Charlson Comorbidity Index	1.31 (1.08–1.59)	.006*	1.30 (1.07–1.58)	.009*
Preoperative hemoglobin	0.92 (0.81–1.05)	.207		
ASA score (ref: 1)				
2	3.26 (1.14–9.28)	.027*	3.16 (1.09–9.15)	.034*
3	3.26 (1.11–9.59)	.032*	2.87 (0.96–8.60)	.060
4	1.19 (0.13–11.33)	.879	1.10 (0.11–10.54)	.937
General anesthesia	0.97 (0.60–1.56)	.904		
Duration of surgery (min)	1.00 (0.99–1.01)	.582		
Time from injury to operation (h)	1.00 (1.00–1.00)	.095		

ASA = American Society of Anesthesiologists, BMI = body mass index, CI = confidence interval, OR = odds ratio.

* *P* ≤ .05 was considered to indicate statistical significance.

## 4. Discussion

Geriatric hip fractures are prevalent in the elderly population and are associated with a high mortality rate. Studies have shown an association between BMI and conditions associated with fractures.^[20,21]^ According to De Laet et al,^[21]^ a lower BMI is associated with an increased risk of all fractures, particularly hip fractures, which depend on bone mineral density. Patients with a BMI of 18.5 kg/m² had a 1.95 times higher risk of hip fractures than those with a BMI of 25 kg/m². Conversely, when comparing BMI 30 kg/m² with BMI < 18.5 kg/m², there was a 17% reduction in the risk of hip fractures. This positive association between body weight and bone mineral density is further supported by Tang et al,^[22]^ who suggested that patients with lower BMI may experience greater bone loss in old age due to lower bone mineral levels, whereas patients with higher BMI may have higher bone mineral density due to various factors, such as mechanical loading, hormones, and higher serum adipokines. Although the association between BMI and hip fractures has been consistently studied, research on postoperative mortality associated with hip fracture surgery according to BMI and subsequent treatment strategies is lacking. Therefore, the establishment of postoperative treatment strategies based on BMI is a direction for future research.

In a study by Chung et al^[23]^ in South Korea, they applied the WHO BMI classification to categorize hip fracture patients into normal (18.5–24.9 kg/m²), overweight and obese (BMI ≥ 25 kg/m²) groups. In their study, the overweight group did not show a significant difference in mortality, but the obese group exhibited significantly lower mortality. Therefore, our study aimed to investigate whether there was a significant difference in mortality between the overweight/obese group, defined as BMI ≥ 25 kg/m² and the normal group, defined as BMI 18.5 to 24.9 kg/m². However, unlike in the underweight group, we did not find a significant difference between the two groups. Being underweight was linked to an increased risk of in-hospital mortality and 1-year mortality.

In this study, apart from BMI, factors such as male sex, CCI, and ASA were associated with 1-year mortality, and factors including male sex, CCI, general anesthesia, and the time from injury to surgery were associated with in-hospital mortality. CCI is used to assess the severity of a patient’s comorbidities and is a predictor of mortality risk, reflecting the patient’s health status.^[24]^ Several studies^[25,26]^ have demonstrated the utility of the CCI as a predictor of 1-year mortality not only in other diseases but also in hip fracture patients. Similarly, CCI was a predictor of in-hospital and 1-year mortality in our study.

Although hip fractures occur more frequently in women, the mortality rate is higher in men.^[27–31]^ Consistent with previous studies,^[27–31]^ our research also demonstrated a higher postoperative mortality rate in men than in women. Although the reasons why geriatric hip fractures lead to higher mortality rates in men are not yet fully understood, Endo et al^[30]^ suggested that men have a higher risk of postoperative complications, such as pneumonia, arrhythmia, delirium, and pulmonary thromboembolism. Furthermore, Wehren et al^[31]^ demonstrated that postoperative infections are associated with higher mortality in women than in men following hip fracture surgery, suggesting that postoperative infections may contribute to the sex disparity in mortality rates after hip fracture surgery. The findings of previous research^[31,32]^ indicate that men tend to have more preoperative comorbidities than women, which also supports this difference in mortality rates.

Various studies on the mortality rates of general versus regional anesthesia in hip fracture surgery have yielded conflicting results. Whereas some studies^[33,34]^ reported higher mortality rates in patients who received general anesthesia, others^[35,36]^ found no significant difference in mortality between the 2 groups. However, Perlas et al^[33]^ indicated that regional anesthesia may lead to better clinical outcomes than general anesthesia in patients undergoing elective total joint replacement. These results suggest a lower incidence of thromboembolic events, major blood loss, and transfusion requirements under regional anesthesia than under general anesthesia. These findings, including those of our study, underscore the importance of considering the anesthesia method in preoperative planning for hip fracture surgery.

Other studies^[37,38]^ have also shown that time to surgery affects the mortality rate after hip fracture surgery. Similar to previous studies,^[37,38]^ our study found that early surgery is associated with reduced mortality rates. Moja et al^[38]^ found that surgery within 48 hours of admission was associated with lower all-cause mortality, consistent with the results of Simunovic et al^[37]^ and Moja et al^[38]^ emphasized that delayed surgery increases the risk of pulmonary, skin, and urinary infections. Therefore, they stressed that preoperative consultation, even if necessary for medical conditions such as cardiac or renal failure, should be conducted within 24 to 48 hours of admission to reduce the risk of complications. Furthermore, the American Academy of Orthopaedic Surgeons guidelines on hip fracture management^[39]^ state that there is moderate evidence supporting better outcomes with hip fracture surgery within 48 hours of admission. Seong et al^[40]^ emphasized the importance of not delaying surgery owing to blood tests or correctable medical conditions based on these studies and guidelines.

Although our study showed no difference in mortality between the overweight and normal weight groups, obesity is usually considered a risk factor in preoperative assessment. Obesity not only increases the risk of medical conditions such as diabetes, coronary artery disease, hypertension, and hyperlipidemia,^[41]^ it also leads to surgical complications such as surgical site infection and acute kidney injury, delaying recovery.^[42]^ Hopf et al^[42]^ explains this by the excess subcutaneous fat tissue leading to a low regional perfusion and oxygen tension state, hindering healing, and increasing the risk of postoperative wound infection due to prolonged operation time.^[43]^ However, recent studies have shown that high BMI increases the risk of post-orthopedic surgery complications such as acute kidney injury compared to normal BMI,^[44]^ whereas mortality is lower, indicating an “obesity paradox.”^[45]^ A recent study showed that obese patients who underwent hip fracture surgery had lower early and late mortality rates than normal-weight patients.^[10]^ This can be explained by the protective effect of sufficient adipose tissue in hypermetabolic-catabolic conditions,^[46]^ such as acute kidney injury or postoperative state.^[47,48]^ However, similar to our findings, another study^[49]^ showed no significant difference in mortality between normal-weight and overweight or obese patients undergoing hip fracture surgery, thus refuting the “obesity paradox” in this context. Also, a study^[9]^ demonstrated that low-weight and morbidly obese patients had the highest mortality rates among other BMI groups. Further studies are required to elucidate this mechanism.

Studies^[9,50]^ have shown that underweight patients have higher complication and mortality rates after hip fracture surgery than other BMI groups. According to Coin et al,^[51]^ underweight elderly patients commonly have malnutrition, osteoporosis, and sarcopenia, which negatively affect overall recovery. Meesters et al^[52]^ explained delayed fracture recovery in patients with malnutrition due to the lack of proteins necessary for fracture healing, leading to prolonged hospitalization and immobility, which can increase the risk of thrombotic complications,^[53]^ a major cause of death in hip fracture patients.^[54]^ Malafarina et al,^[55]^ as mentioned above, also found that low BMI, in terms of malnutrition, is associated with higher mortality in hip fracture patients. They emphasized the importance of a low BMI as a significant measure for evaluating malnutrition, despite its various limitations, as it can serve as a simple and repetitive indicator of nutritional status. In addition to the nutritional implications and resulting complications, underweight patients also have lower respiratory muscle strength and immune function than other patient groups, leading to a higher risk of respiratory complications.^[56]^ According to a cohort study,^[57]^ underweight patients had a higher risk of pneumonia and the need for intensive care unit admission than other BMI groups. This could be another factor that contributes to the higher mortality rate observed in underweight patients undergoing hip fracture surgery. To address this, it is essential to meticulously assess the nutritional status of underweight patients and refer them to a nutrition support team to implement individualized nutritional support and treatment strategies based on their condition. For patients at high risk of malnutrition, if preoperative oral diets are insufficient, enteral nutrition can be provided, and additional total parenteral nutrition can be considered. Through these measures, preoperative and postoperative nutritional support strategies for underweight patients are expected to reduce the incidence of complications and mortality.

This study demonstrated that the underweight group had a higher postoperative mortality rate than the other BMI groups among elderly patients with hip fractures. Therefore, preoperative risk assessment and postoperative management in elderly underweight patients are critical. Consequently, future research should focus on interventions, such as nutrition, drugs, and other strategies to reduce the incidence of complications and mortality in underweight patients. For instance, Hirsch et al^[58]^ highlighted that preoperative nutrition to meet the demands of the stress state in orthopedic surgery, reduce loss of lean mass, and promote nitrogen balance decreased hospitalization costs, complications, and length of stay. Moreover, for surgeries requiring physical rehabilitation, such as hip fracture surgery, several studies have shown that nutritional management during exercise/rehabilitation sessions can expedite the return of daily functional level and strength.^[59–61]^ Another study^[62]^ conducted in the UK demonstrated that not only functional aspects but also mortality after hip fracture could be improved through nutritional support.

This study had some limitations. First, the composition of body components, such as muscles and fat, was not directly reflected because BMI was used for patient classification. Second, since only overall mortality was investigated, regardless of the cause of death, it was impossible to clearly establish the causality between BMI and mortality more than 1 year after surgery. Third, the average time from injury to surgery is relatively long. This extended time from injury to operation, averaging 134.5 hours, observed in our study can be primarily attributed to the institutional protocols previously in place at our hospital. Historically, we recommended comprehensive cardiac and pulmonary evaluations, including transthoracic echocardiography and pulmonary function tests, for elderly hip fracture patients prior to surgery. These evaluations, while thorough, contributed to the longer preoperative waiting periods. However, we have recently revised our approach. Our current practice aims to expedite surgical intervention, targeting surgery within 48 hours after injury. This shift excludes only those patients with significantly high cardiac or pulmonary risks who necessitate detailed evaluations. This change in protocol reflects our commitment to improving patient outcomes by reducing delays in surgical treatment.

Despite these limitations, our study has several strengths. First, its findings were based on a single-center design targeting patients operated on by a single surgeon, excluding the effects of surgical skill and hospital capabilities. In addition, we included a sufficient number of patients to obtain statistically significant results. Finally, the majority of patients were of a single ethnicity, Korean, which excluded racial differences.

This study was conducted to demonstrate the association between BMI and postoperative mortality in patients after hip fracture surgery and to provide new insights not only into the establishment of patient management strategies based on BMI after hip fracture surgery but also into the prevention and management of high-risk patients, such as elderly and underweight patients.

## 5. Conclusion

Our study highlights the importance of BMI in predicting postoperative mortality in elderly patients undergoing hip fracture surgery. Underweight individuals have a notably higher risk of in-hospital and 1-year mortality than normal or overweight patients. These results highlight the need for thorough preoperative risk assessment and tailored postoperative care, especially for high-risk groups, such as the elderly and underweight individuals. Future research should concentrate on interventions, such as nutritional support, preoperative optimization, and enhanced postoperative care, to improve outcomes in vulnerable populations.

## Author contributions

**Conceptualization:** Suk-Kyoon Song.

**Data curation:** Chan-Hee Park, Seung-Hoon Lee, Suk-Kyoon Song.

**Formal analysis:** Chan-Hee Park, Seung-Hoon Lee, Rim Lee, Dong-Young Kim.

**Funding acquisition:** Suk-Kyoon Song.

**Investigation:** Dong-Young Kim.

**Methodology:** Chan-Hee Park, Rim Lee, Dong-Young Kim, Suk-Kyoon Song.

**Supervision:** Myung-Rae Cho.

**Validation:** Suk-Kyoon Song.

**Writing – original draft:** Chan-Hee Park, Seung-Hoon Lee, Suk-Kyoon Song.

**Writing – review & editing:** Rim Lee, Dong-Young Kim, Myung-Rae Cho, Suk-Kyoon Song.
